# The effect of interbrain synchronization in gesture observation: A fNIRS study

**DOI:** 10.1002/brb3.1663

**Published:** 2020-05-29

**Authors:** Giulia Fronda, Michela Balconi

**Affiliations:** ^1^ Department of Psychology Catholic University of Milan Milan Italy; ^2^ Research Unit in Affective and Social Neuroscience Catholic University of Milan Milan Italy

**Keywords:** fNIRS, gestures, interbrain connectivity, observation, valence

## Abstract

**Introduction:**

Gestures characterize individuals' nonverbal communicative exchanges, taking on different functions. Several types of research in the neuroscientific field have been interested in the investigation of the neural correlates underlying the observation and implementation of different gestures categories. In particular, different studies have focused on the neural correlates underlying gestures observation, emphasizing the presence of mirroring mechanisms in specific brain areas, which appear to be involved in gesture observation and planning mechanisms.

**Materials and methods:**

Specifically, the present study aimed to investigate the neural mechanisms, through the use of functional Near‐Infrared Spectroscopy (fNIRS), underlying the observation of affective, social, and informative gestures with positive and negative valence in individuals' dyads composed by encoder and decoder. The variations of oxygenated (O2Hb) and deoxygenated (HHb) hemoglobin concentrations of both individuals were collected simultaneously through the use of hyperscanning paradigm, allowing the recording of brain responsiveness and interbrain connectivity.

**Results:**

The results showed a different brain activation and an increase of interbrain connectivity according to the type of gestures observed, with a significant increase of O2Hb brain responsiveness and interbrain connectivity and a decrease of HHb brain responsiveness for affective gestures in the dorsolateral prefrontal cortex (DLPFC) and for social gestures in the superior frontal gyrus (SFG). Furthermore, concerning the valence of the observed gestures, an increase of O2Hb brain activity and interbrain connectivity was observed in the left DLPFC for positive affective gestures compared to negative ones.

**Conclusion:**

In conclusion, the present study showed different brain responses underlying the observation of different types of positive and negative gestures. Moreover, interbrain connectivity calculation allowed us to underline the presence of mirroring mechanisms involved in gesture‐specific frontal regions during gestures observation and action planning.

## Graphical abstract

The present study aimed to investigate the neural mechanisms, through the use of functional Near‐Infrared Spectroscopy (fNIRS), underlying the observation of affective, social, and informative gestures with positive and negative valence in individuals' dyads composed by encoder and decoder. The variations of oxygenated (O2Hb) and deoxygenated (HHb) hemoglobin concentrations of both individuals were collected simultaneously through the use of hyperscanning paradigm, allowing the recording of brain responsiveness and interbrain connectivity. The present study showed different brain responses underlying the observation of different types of positive and negative gestures.

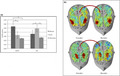

## 1 INTRODUCTION

Gestures are configured as a communicative vehicle that characterizes verbal and nonverbal communication (McNeill, 1992). Studies in the psychological, social, and linguistic fields have been interested in the investigation of gestures trough different perspectives, analyzing the relationship between thought, language, and action (Kong, Law, Kwan, Lai, & Lam, 2015). On the contrary, studies in the neuroscientific field have been interested in observing the neural correlates underlying the perception and the implementation of gestures with different functions (Bates & Dick, 2002; Green et al., 2009; Willems & Hagoort, 2007). Action observation, recognition, and interpretation, indeed, appears to be a fundamental ability for communication and social perception processes (Chong, Williams, Cunnington, & Mattingley, 2008).

In particular, several neuroimaging studies have investigated the neural correlates underlying the observation and the reproduction of different gesture categories (Chong et al., 2008; Molnar‐Szakacs, Wu, Robles, & Iacoboni, 2007; Mühlau et al., 2005), highlighting the presence of different brain areas, such as the frontal and intraparietal cortex and the dorsal and ventral premotor cortex (PMC), which constitute a wide network implicated in the observation (Caspers, Zilles, Laird, & Eickhoff, 2010; Molenberghs, Cunnington, & Mattingley, 2012) of different types of gestures: familiar or not (Liew, Han, & Aziz‐Zadeh, 2011), significant and not significant (Lui et al., 2008; Newman‐Norlund, van Schie, van Hoek, Cuijpers, & Bekkering, 2010) and directed or not to an object (Decety et al., 1997; Grèzes, Costes, & Decety, 1999; Villarreal et al., 2008). In addition to this extensive neural network, other studies have demonstrated, through the use of different methodologies (Buccino et al., 2001; Filimon, Nelson, Hagler, & Sereno, 2007; Kilner, Neal, Weiskopf, Friston, & Frith, 2009; Mukamel, Ekstrom, Kaplan, Iacoboni, & Fried, 2010; Pokorny et al., 2015; Rizzolatti & Fogassi, 2014), the involvement of the fronto‐parietal network in action observation, highlighting the presence of mirroring mechanisms in these cerebral regions (Crivelli, Sabogal Rueda, & Balconi, 2018; Gallese, Fadiga, Fogassi, & Rizzolatti, 1996; di Pellegrino, Fadiga, Fogassi, Gallese, & Rizzolatti, 1992; Rizzolatti, Fadiga, Gallese, & Fogassi, 1996).

Mirroring mechanisms, in particular, allow the visual input involved in the observed motor act to reach and activate the same fronto‐parietal circuits involved in the same action execution (Nelissen & Vanduffel, 2011), allowing individuals, thanks to a representational level, to plan their actions (Freedberg & Gallese, 2007; Gallese, 2006) and to understand the meaning of observed actions (Rizzolatti & Craighero, 2004; Rizzolatti, Fogassi, & Gallese, 2001), creating a direct link between action observation and execution (Holle, Gunter, Rüschemeyer, Hennenlotter, & Iacoboni, 2008; Huxham, Dick, & Stringer, 2009).

Moreover, the fronto‐parietal mirror neuron and other brain structures, such as the posterior inferior frontal gyrus, the precentral gyrus, and the rostral part of the inferior parietal lobule, appear to be involved in mirroring mechanisms, (Lepage & Théoret, 2006) and socially relevant functions and processes, such as empathy (Carr, Iacoboni, Dubeaut, Mazziotta, & Lenzi, 2003; Molnar‐Szakacs et al., 2007), intention comprehension (Iacoboni et al., 2005; Molnar‐Szakacs et al., 2007), and communication (Iacoboni et al., 2005; Molnar‐Szakacs et al., 2007), leading individuals involved in the exchange to develop greater resonance and interbrain coupling mechanisms (Balconi & Vanutelli, 2017; Lindenberger, Li, Gruber, & Müller, 2009; Vanutelli, Nandrino, & Balconi, 2016).

Specifically, interbrain coupling or connectivity can be defined as the correlation between two time series (Friston, 2011) which reflects the agents' neuronal activations (Balconi, Crivelli, & Vanutelli, 2017; Chaudhary, Hall, DeCerce, Rey, & Godavarty, 2011) providing information about neuropsychological events spatially remote. In particular, interbrain connectivity, allowing the simultaneous recording of brain activity during joint actions execution, provides information about interpersonal coupling dynamics, mechanisms of social comprehension (Balconi & Vanutelli, 2017; Crivelli & Balconi, 2017; Knoblich, Butterfill, & Sebanz, 2011) and synchronic mechanisms underlying gestural communication (Balconi & Pagani, 2015; Hasson, Ghazanfar, Galantucci, Garrod, & Keysers, 2012; Liu, Saito, & Oi, 2015; Vanutelli et al., 2016).

In light of this evidence, in the present study, in order to investigate the brain correlates underlying the observation of different positive and negative types of gestures (affective, social, and informative), the neural responses of encoders and decoders were recorded through the use of fNIRS in hyperscanning, that is a very effective neuroimaging technique for the recording of individuals' neural activity underlying emotional or social processes (Balconi & Cortesi, 2016; Balconi, Vanutelli, & Grippa, 2017; Crivelli et al., 2018) under natural or maximally ecological conditions (Balconi & Molteni, 2016; Crivelli & Balconi, 2017), providing information on interbrain tuning and “resonance” and implicit coupling mechanisms (Balconi, Gatti, & Vanutelli, 2018; Balconi & Vanutelli, 2017; Vanutelli et al., 2016).

Specifically, the present study aimed to observe possible differences in individuals' neural responses underlying the observation of different types of gestures: affective, social, and informative of different valence: positive and negative.

In particular, affective gestures are aimed to express their moods and share their emotional experiences with the interlocutor (Tomasello, Carpenter, Call, Behne, & Moll, 2005).

On the contrary, social gestures are aimed at managing interpersonal relationships and are useful for starting, maintaining or interrupting an interaction with another individual (Kendon, 2017), providing the implementation of inclusion, cooperation, and exclusion behaviors, that can elicit positive and negative emotions in the interlocutor (Bavelas, Chovil, Lawrie, & Wade, 1992; Bressem & Müller, 2017; Calbris, 2011).

Finally, informative gestures are aimed at communicating a physical state to the interlocutor with the purpose to direct the decoder attention toward a specific element (Enfield, 2009; Enfield, Kita, & de Ruiter, 2007), satisfying different communication functions that can provide positive and negative emotional experiences (Enfield, 2009; Enfield et al., 2007).

Regarding this different types of gestures, several studies (Bush, Luu, & Posner, 2000; Carter et al., 1998; Craig & Craig, 2009; Critchley, Wiens, Rotshtein, Öhman, & Dolan, 2004) have observed the neural correlates underlying affective, social, and informative gestures. In particular, affective and social gestures result to activate some cerebral structures, such as the anterior cingulate cortex (ACC) and the insular cortex, that are more involved in emotional regulation. Moreover, insular cortex appears to be involved in empathic processes, body representation, and emotional experience (Bush et al., 2000; Carter et al., 1998; Craig & Craig, 2009; Critchley et al., 2004) presenting connections with other structures such as the orbitofrontal cortex (OFC), the DLPFC and the anterior cingulate cortex (ACC) (Mufson & Mesulam, 1982; Viskontas, Possin, & Miller, 2007). In addition to the insular cortex, some subcortical structures, such as the amygdala, also play a fundamental role in the emotional experience (Adolphs, 2002; Dolan, 2002; Zald, 2003).

Despite the role of this subcortical structure in emotional processes, our interest focuses mainly on the cortical regions involved in cognitive and emotional processes, since fNIRS measures cortical neuronal firing through the hemodynamic changes due to neurovascular coupling (Curtin et al., 2019; Fuster et al., 2005; Heeger & Ress, 2002).

In this regard, as demonstrated by previous studies (Blair, Morris, Frith, Perrett, & Dolan, 1999; Wildgruber et al., 2004), affective and social gestures result to activate more the frontal regions, such as the medial part of the ventral prefrontal cortex and the DLPFC, that is implicated in emotional valence of more expressive and emotional gestures.

On the contrary, other types of gestures, such as informative ones, that are involved in attentional shift and gaze perception of hand movements or hand gestures, appear to activate posterior regions, such as the parietal lobule and the superior temporal sulcus (STS) (Nakamura et al., 2004; Pelphrey, Morris, & Mccarthy, 2004; Thompson, Hardee, Panayiotou, Crewther, & Puce, 2007; Wheaton, Thompson, Syngeniotis, Abbott, & Puce, 2004). In the light of this evidence, we expected to observe an increase of O2Hb and a decrease of HHb activity in frontal areas according to the observation of affective and social gestures, which appear to be more involved in individuals' emotional responses and social processes (Bavelas et al., 1992; Bressem & Müller, 2017; Calbris, 2011; Müller, 2016; Tomasello et al., 2005). In particular, during the observation of affective gestures, we expected to observe an increase of O2Hb and a decrease of HHb activity in DLPFC, which appears to be more involved in mind theory processes, interpersonal relationships, other people's states understanding (Bavelas et al., 1992; Bressem & Müller, 2017; Calbris, 2011; Kendon, 2017; Müller, 2004, 2016) and attentional processing of emotional information (Fragopanagos, Kockelkoren, & Taylor, 2005; Liotti & Mayberg, 2001) considering its role in top‐down attentional control (MacDonald, Cohen, Stenger, & Carter, 2000; Vanderhasselt, De Raedt, Baeken, Leyman, & D'haenen, 2006).

Instead, during the observation of social gesture, we expected to observe an increase of O2Hb activity in SFG area, which appears to be more involved in emotional regulation and interaction and social understanding mechanisms (Baker, Liu, et al., 2016; Kalbe et al., 2010; Liu et al., 2015; Suzuki, Niki, Fujisaki, & Akiyama, 2011). Related to informative gestures, instead, we expected to observe an increase of O2Hb activity in parietal areas, that are more involved in visual and sensorimotor integration processes and in the imagination of body in time and space (Janowski, Kurpas, Kusz, Mroczek, & Jedynak, 2013; Nicolle et al., 2012; Ruby & Decety, 2001).

Moreover, considering gestures valence (positive, negative), we expected to observe a different cerebral asymmetry in the DLPFC area, more involved in interpersonal and emotional processes (Bavelas et al., 1992; Bressem & Müller, 2017; Calbris, 2011; Kendon, 2017; Müller, 2004, 2016) according to the observation of positive and negative affective gestures, which are those more involved in emotional and affective processes communication (Tomasello, Carpenter, & Liszkowski, 2007). In particular, based on neural signatures of affective experience model (Balconi, Grippa, & Vanutelli, 2015; Davidson, 1992), that postulates that positive stimuli more activate left frontal areas compared with negative ones that more activate right frontal side, we expected to observe an increase of O2Hb in the left DLPFC side during the observation of positive affective gestures, that induce positive emotions and approaching and sharing behaviors in individuals. On the contrary, we expected to observe an increase of O2Hb activity in the right DLPFC side during the observation of negative affective gestures, inducing withdrawal behavior.

Finally, thanks to the use of fNIRS in hyperscanning, which allows the simultaneous recording of the activity of the two interagents individuals, we expected to observe an increase of interbrain connectivity and resonance mechanisms in the frontal areas during the observation of affective and social gestures. In particular, we expected to observe an increase of interbrain connectivity in encoder and decoder in frontal areas during the observation of affective and social gestures and in parietal areas for informative one due to the presence of mirroring mechanisms, that are activated during action observation, imagination and planning, and in line with the specificity of these brain areas in response to gesture types. Indeed, frontal areas are more involved in relational, prosocial, and empathic processes (Balconi & Bortolotti, 2012, 2013; Balconi, Falbo, & Conte, 2012; Rameson & Lieberman, 2009), while parietal ones are more implicated in processes concerning gestures' observation and execution (Caplan, 2003; Ekstrom et al., 2005; Jones & Wilson, 2005; Sirota et al., 2008).

Starting from this evidence, we expected to observe a similar neural activation in the encoder, who observed the gesture and mentally plans the action to be successively reproduced, and in the decoder, who only observed the gesture without any action reproduction.

Indeed, as demonstrated by previous studies, mirroring processes create a direct link between gestures' observation and execution in both the actor who is required to successively reproduce the action and who has simply to observe the action itself (Holle et al., 2008; Huxham et al., 2009) because actions observation activates the same brain areas involved in that actions execution.

## 2 METHODS

### 2.1 Participants

For the research conduction, seventeen dyads of participants (*M*_age_ = 26.98; *SD*_age_ = 0.03) of the same gender were recruited, for a total of 34 subjects. In particular, 14 dyads were composed of participants of female gender, while participants of male gender composed three dyads. Recruited participants were university students. Specifically, the participants, coupled in dyads, did not know each other. Then, one of each dyads' participants was randomly assigned the role of encoder or decoder, who were asked to perform different functions. Participants were recruited with the following criteria: age between 18 and 40 years and normal or corrected‐to‐normal visual acuity, no neurological or cognitive deficits. Participants gave their voluntary consent to participate in the research after signing the informed consent. The local ethics committee of the Department of Psychology of the Catholic University of the Sacred Heart of Milan was approved by the principles and guidelines of the Helsinki Declaration.

### 2.2 Procedure

For the conduct of the experiment, participants were invited to sit in a room at a distance of 60 cm from a centrally placed computer that allows observing the videos reproducing different gesture types, presented through the E‐Prime 2.0 software (E‐prime2 software; Tools Psychology Software Inc.). Specifically, 60 videos, that reproduced a non‐verbal interaction between two actors, characterized by different gesture types (affective, social, and informative with positive and negative valence), were shown to participants. The presentation of the 60 videos took place in three randomized blocks, each consisting of 20 stimuli, with an interval of a few minutes to prevent participants' fatigue. The 60 videos consist in the reproduction of: 10 affective gestures with positive valence, aimed at communicating to the interlocutor a state of well‐being, 10 affective gestures with negative valence, aimed of transmitting a state of malaise, 10 social gestures with positive valence, aimed at starting or maintaining a relationship with the interlocutor, 10 social gestures with negative valence, aimed at interrupting the relationship with the interlocutor, 10 informative gestures with positive valence and 10 informative gestures with negative valence, aimed to direct the attention of the interlocutor toward a specific object in the environment. The valence of informative gestures was defined by the context that was introduced before gesture video presentation.

**FIGURE 1:**
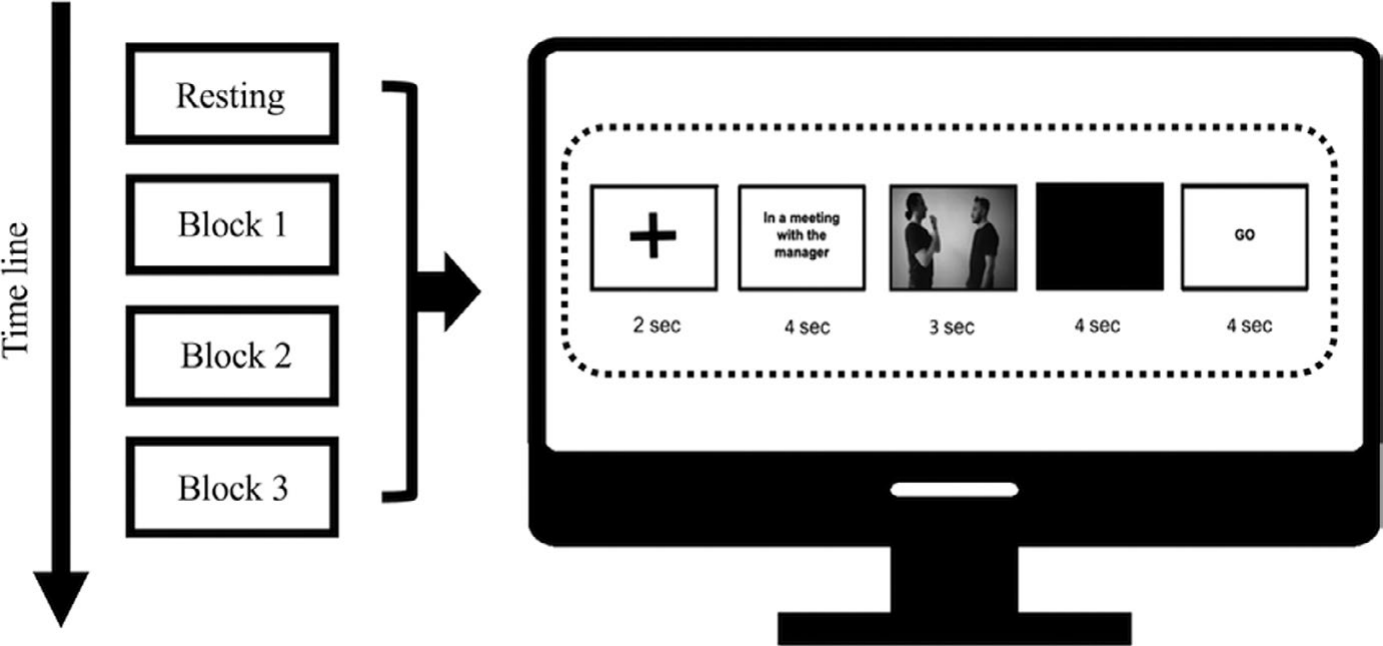
Experimental procedure of the task administered to participants

Specifically, the experiment required both dyads participants firstly to observe the videos that appeared on the screen for a duration of 3 s (sec.). Subsequently, either one participant, casually identified as the encoder, was asked to reproduce the gesture observed toward his companion, the decoder. Specifically, the experiment was carried out in the following way: an initial phase of task familiarization, followed by the execution of the three task blocks (order randomized). The administration of the task consists of the presentation of a 2 s black screen; the presentation of a slide containing a context sentence, lasting 4 s, to help individuals to understand the meaning of gesture presented; the appearance of the video reproducing the gesture to be observed for 3 s; the presentation of a 4 s black screen and the presentation of a slide with the “go” signal to indicate participants to reproduce the gesture (Figure 1).

Fourteen judges (seven males and seven females) were recruited (*M*_age_ = 28.34, *SD*_age_ = 0.04) for the stimuli validation using a Likert scale of seven points. In particular, the evaluation concerns some gestures features, such as commonality, frequency of use, complexity, social meaning, familiarity, and emotional impact for the three types of gesture (affective, social, and informative). All gestures were homogeneous for the previous mentioned characteristics that were verified by statistical analysis, differing only for emotional degree and social content that differently characterize affective, social, and informative gestures. For the stimuli categories, statistical analysis was used to verify the similarity for previous characteristics (for all comparisons *p* ≥ .12). In particular, from statistical analysis emerged a difference only in terms of emotional degree (for social type *M* = 5.34, *SD* = 0.04; for affective *M* = 5.99, *SD* = 0.03; for informative *M* = 3.41, *SD* = 0.04) and social content (for social type *M* = 5.87, *SD* = 0.02; for affective *M* = 4.78, *SD* = 0.03; for informative *M* = 3.58, *SD* = 0.02).

### 2.3 fNIRS recording and signal processing

A NIRScout system (NIRx Medical Technologies, LLC) with a 16‐optode matrix was used to record hemodynamic responses consisting of the variation of O2Hb and HHb concentrations. Specifically, through the use of an ElectroCap, eight sources and eight detectors were placed on each scalp following the 10/5 international system (Oostenveld & Praamstra, 2001).

**FIGURE 2:**
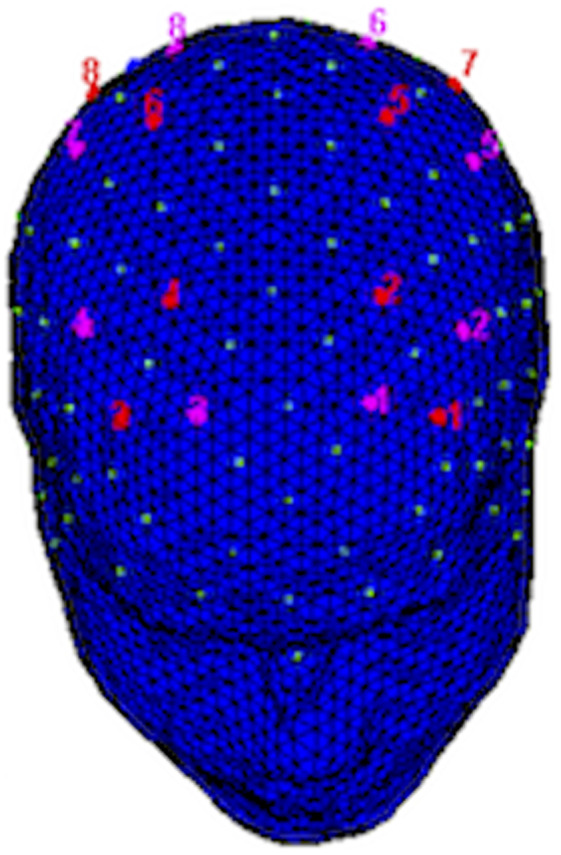
Location of the sources (red) and detectors (violet) of fNIRS montage. In particular, sources are located in the following positions: F3‐FC1, F4‐FC2, CP1‐P3, CP2‐P4 and detectors as follows: F1‐FC3, F2‐FC4, CP3‐P1, CP4‐P2

The distance between sources and detectors was kept at 30 mm for contiguous optodes and a near‐infrared light of two wavelengths (760 and 850 nm) was used. Specifically, the eight sources were positioned in the following positions: F3‐FC1, F4‐FC2, CP1‐P3, CP2‐P4; while the eight detectors were placed as follows: F1‐FC3, F2‐FC4, CP3‐P1, and CP4‐P2 (Figure 2). The optodes' placement resulted in acquiring the following channels: Ch1 (F3‐F1), Ch2 (F3‐FC3), Ch3 (FC1‐F1), Ch4 (FC1‐FC3), Ch5 (F4‐F2), Ch6 (F4‐FC4), Ch7 (FC2‐F2), Ch8 (FC2‐FC4), Ch9 (CP1‐CP3), Ch10 (CP1‐P1), Ch11 (P3‐CP3), Ch12 (P3‐P1), Ch13 (CP2‐CP4), Ch14 (CP2‐P2), Ch15 (P4‐CP4), and Ch16 (P4‐P2).

Before the task beginning, a preliminary baseline of 120 s was recorded. The variation in the concentration of O2Hb and HHb hemoglobin was continuously recorded based on channels wavelength and position. The sampling rate was set to 6.25 Hz. For the signal processing, nirsLAB software was used (v2014.05; NIRx Medical Technologies LLC). A 0.01–0.3 Hz bandpass filter was used for O2Hb and HHb data filtering (Oda, Sato, Nambu, & Wada, 2018). Raw time series were visually inspected to detect noisy channels (e.g., due to large motion errors, sudden amplitude changes, poor coupling), excluding channels with a poor optical coupling, for example, absence of the ~1 Hz heartbeat oscillations in raw signals (Pinti et al., 2015).

O2Hb and HHb mean concentration for each channel was calculated for gesture category (affective, social, and informative), and valence (positive and negative). The mean concentration of each channel was computed by averaging data across the trials, starting from the appearance of the video reproducing the gesture to be observed for the following 3 s. For each channel and participant, according to the mean concentrations in the time series, the effect size in every block was calculated as the difference of the means of the gesture observation steps (m2) and the baseline (m1) divided by the standard deviation (*SD*) of the baseline: *d* = (m2‐m1)/*SD* (Cohen's *d* value).

This normalized's index's average can be calculated despite the unit since the effect size parameter is not influenced by the differential pathlength factor (DPF), overcoming the fact that fNIRS raw data were initially related values and could not be precisely measured across participants or channels (Matsuda & Hiraki, 2006; Schroeter, Zysset, Kruggel, & Von Cramon, 2003; Shimada & Hiraki, 2006).

### 2.4 Data analysis

Three types of analyses were completed according to O2Hb‐ and HHb‐dependent measures. The first ANOVA was applied to single‐brain data to test the effect of independent measures on O2Hb and HHb concentration for each participant (single‐brain analysis). Secondly, Pearson correlational analysis for each couple of participants of encoder/decoder was calculated for each dependent measure finalizing to compute the synchronization values within each couple for each measure. Thirdly, these indices were put into different ANOVA tests, as dependent variables, in order to evaluate differences in synchrony strength across the experimental conditions (interbrain connectivity analysis).

The degrees of freedom were corrected for all the ANOVAs using Greenhouse‐Geisser epsilon with a 0.05 significance level. Moreover, contrast analyses and multiple comparisons with the Bonferroni test were applied. Finally, data distribution normality was tested with kurtosis and asymmetry tests. Due to multiple comparisons, type I and type II errors were considered and power analysis allowed to support adequate limitation to increasing of these errors.

## 3 RESULTS

### 3.1 Single‐brain analyses

For single‐brain analyses, Role (encoder/decoder), gesture Valence (positive/negative), Lateralization (left/right), gesture Type (social/affective/informative), and Region (four anterior and four posterior) were used as independent measures. Specifically, Region was composed for both left/right homologous sides. In particular, for anterior areas, the values of Ch1 and Ch5 correspond to the left and right frontal eye fields (FEF) activity, the values of Ch2 and Ch6 correspond to the left and right DLPFC activity, the values of Ch3 and Ch7 correspond to the left and right superior frontal gyrus (SFG) activity, the values of Ch4 and Ch8 correspond to the left and right dorsal premotor cortex (DPMC) activity.

Regarding posterior areas, the values of Ch9 and Ch13 correspond to the left and right supramarginal gyrus activity; the values of Ch10 and Ch14 correspond to the left and right superior parietal lobule activity; the values of Ch11 and Ch15 correspond to the left and right angular gyrus activity; the values of Ch12 and Ch16 correspond to the left and right lateral portion of superior parietal lobule activity.

A mixed‐model ANOVA was applied to O2Hb‐ and HHb‐dependent measures. We reported only the significant comparisons.

**FIGURE 3:**
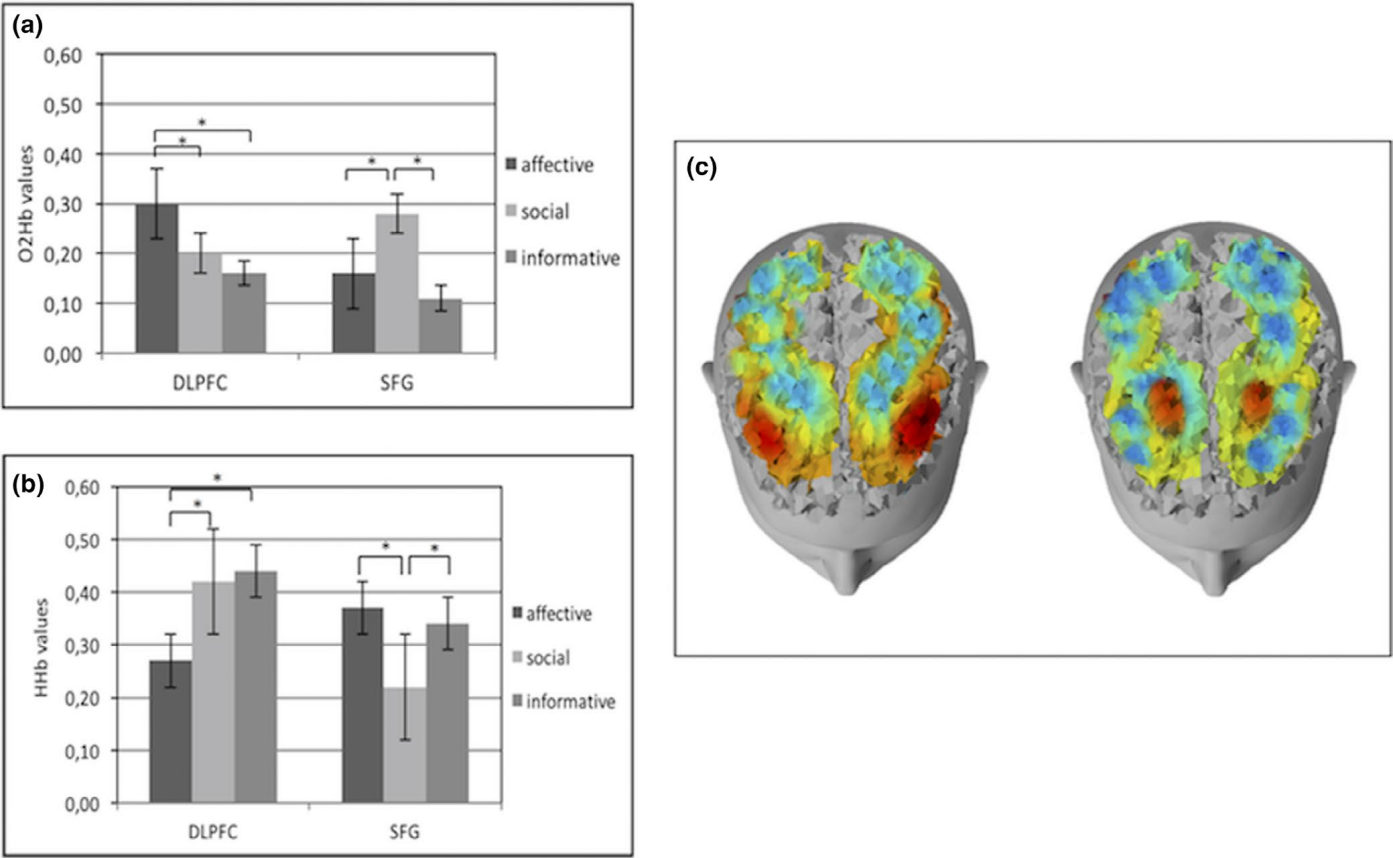
(a) Histogram of O2Hb activity according to the three different gestures types (affective, social, and informative) in DLPFC and SFG. Bars represent ±1*SE*. Stars mark statistically significant (*p*<.05) pairwise comparisons. (b) Histogram of HHb activity according to the three different gestures types (affective, social, and informative) in DLPFC and SFG. Bars represent ±1*SE*. Stars mark statistically significant (*p*<.05) pairwise comparisons. (c) Representation, from left to right, of brain responsiveness for affective and social gestures. The figure shows an increase of O2Hb activity (red color) in DLPFC for affective gestures and SFG for social gestures

**FIGURE 4:**
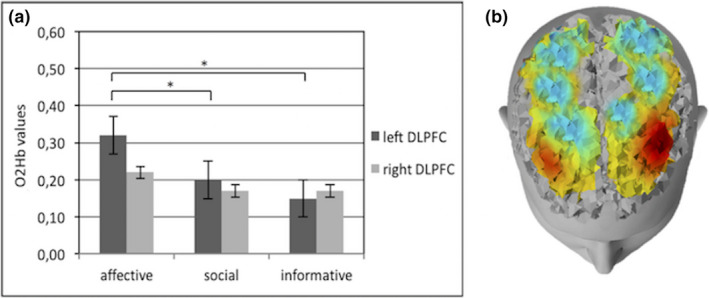
(a) Histogram of O2Hb activity for three different gestures (affective, social, and informative) in the left and right side of the DLPFC. Bars represent ±1*SE*. Stars mark statistically significant (*p*<.05) pairwise comparisons. (b) Representation of O2Hb brain responsiveness for affective gestures in DLPFC left and right side. The red color shows the increase of O2Hb brain responsiveness in the DLPFC left side compared with the right one for affective gestures

Specifically, regarding O2Hb activity, as shown by ANOVA, significant interaction effects for Type x Region (*F*[14,260] = 7.24; *p* < .001; *η* ^2^ = 0.28), and Valence × Lateralization × Region (*F*[7,70] = 8.03; *p* < .001; *η* ^2^ = 0.28) were observed. In particular, by post‐hoc comparisons, an increase of O2Hb brain activity was observed for affective more than social (*F*[1,33] = 8.50; *p* < .001; *η* ^2^ = 0.30) and informative (*F*[1,33] = 7.98; *p* < .001; *η* ^2^ = 0.29) gestures in DLPFC and for social more than affective (*F*[1,33] = 7.76; *p* < .001; *η* ^2^ = 0.28) and informative (*F*[1,33] = 7.92; *p* < .001; *η* ^2^ = 0.29) gestures in SFG (Figure 3a,c). Moreover, as revealed by ANOVA, positive gestures observation showed an increase of O2Hb activity in DLPFC left side responsiveness: this effect was specific for affective more than social (*F*[1,33] = 9.21; *p* < .001; *η* ^2^ = 0.35) and informative (*F*[1,33] = 8.45; *p* < .001; *η* ^2^ = 0.30) gestures (Figure 4a,b).

Regarding HHb, a significant effect has emerged for Type × Region (*F*[7,260] = 10.11; *p* < .001; *η* ^2^ = 0.34). In particular post hoc comparisons showed a decrease of HHb for affective more than social (*F*[1,33] = 7.56; *p* < .001; *η* ^2^ = 0.27) and informative (*F*[1,33] = 7.11; *p* < .001; *η* ^2^ = 0.26) gestures in DLPFC and for social more than affective (*F*[1,33] = 7.98; *p* < .001; *η* ^2^ = 0.28) and informative (*F*[1,33] = 8.04; *p* < .001; *η* ^2^ = 0.30) ones in SFG (Figure 3b,c).

### 3.2 Interbrain connectivity analyses

Considering the O2Hb and HHb concentration raw database, interparticipant correlational indices were calculated to compute the synchronization within each dyad. These indices (*r* values) were successively used as dependent variables in mixed‐model ANOVA tests for O2Hb and HHb, with the following repeated factors: Type, Valence, Lateralization, and Region.

**FIGURE 5:**
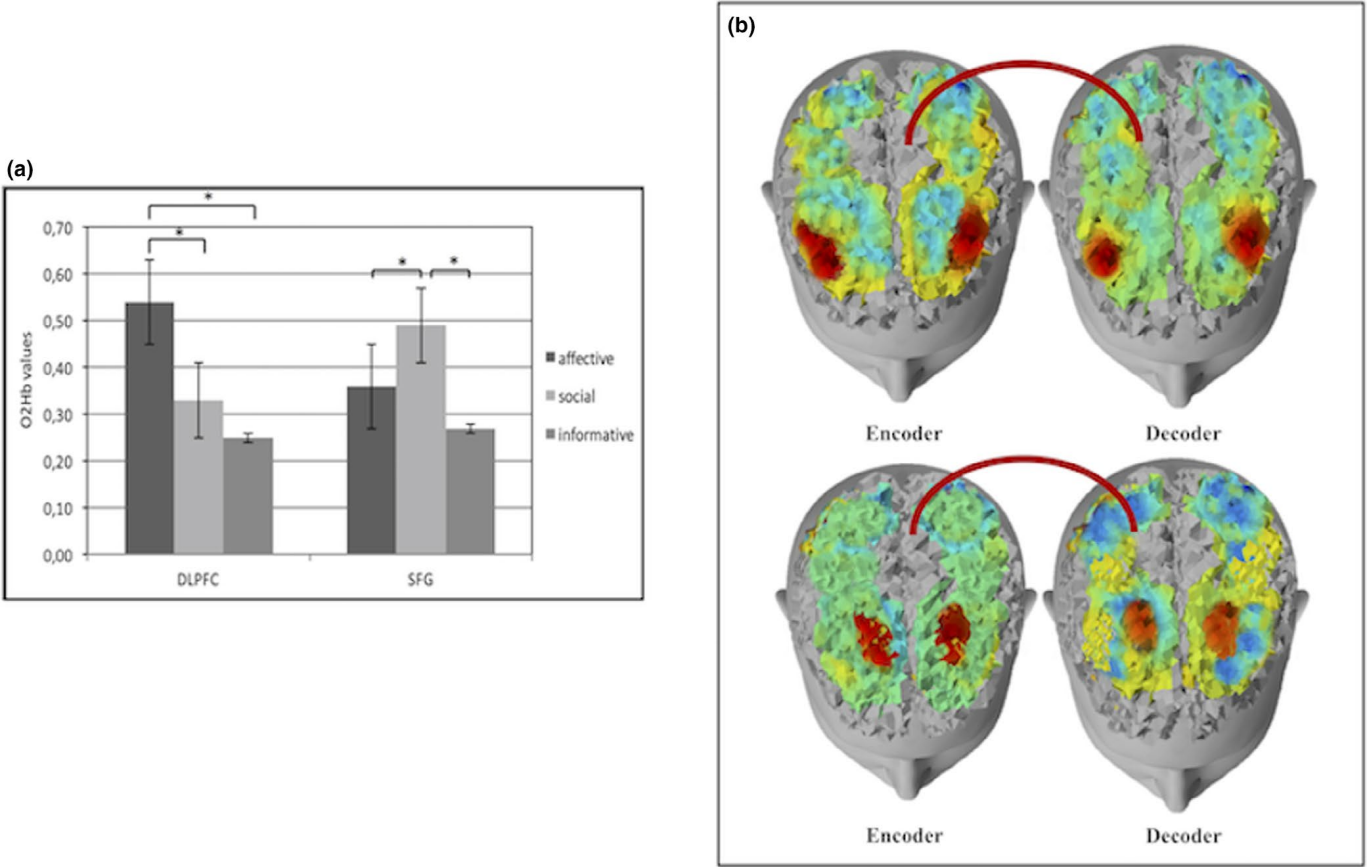
(a) Histogram of O2Hb interbrain connectivity according to the three different gestures types (affective, social, and informative) in DLPFC and SFG. Bars represent +‐1SE. Stars mark statistically significant (*p*<.05) pairwise comparisons. (b) Representation of brain responsiveness for affective and social gestures. The superior figure shows an increase of O2Hb interbrain connectivity (red color) in DLPFC for affective gestures in encoder and decoder and the inferior figure shows an increase of O2Hb interbrain connectivity (red color) in SFG for social gestures in encoder and decoder

From ANOVA, for O2Hb, a significant effect emerged for Type × Region (*F*[2,105] = 7.89; *p* < .001; *η* ^2^ = 0.27) and Valence × Region × Lateralization (*F*[14,215] = 8.01; *p* < .001; *η* ^2^ = 0.31). Specifically, an increase for interbrain synchronization (increased Pearson coefficients) was found for affective gesture in DLPFC than social (*F*[1,16] = 7.89; *p* < .001; *η* ^2^ = 0.27) and informative ones (*F*[1,16] = 7.12; *p* < .001; *η* ^2^ = 0.27) and for social gesture in SFG than affective (*F*[1,16] = 8.03; *p* < .001; *η* ^2^ = 0.27) and informative ones (*F*[1,16] = 7.98; *p* < .001; *η* ^2^ = 0.28; Figure 5a,b).

**FIGURE 6:**
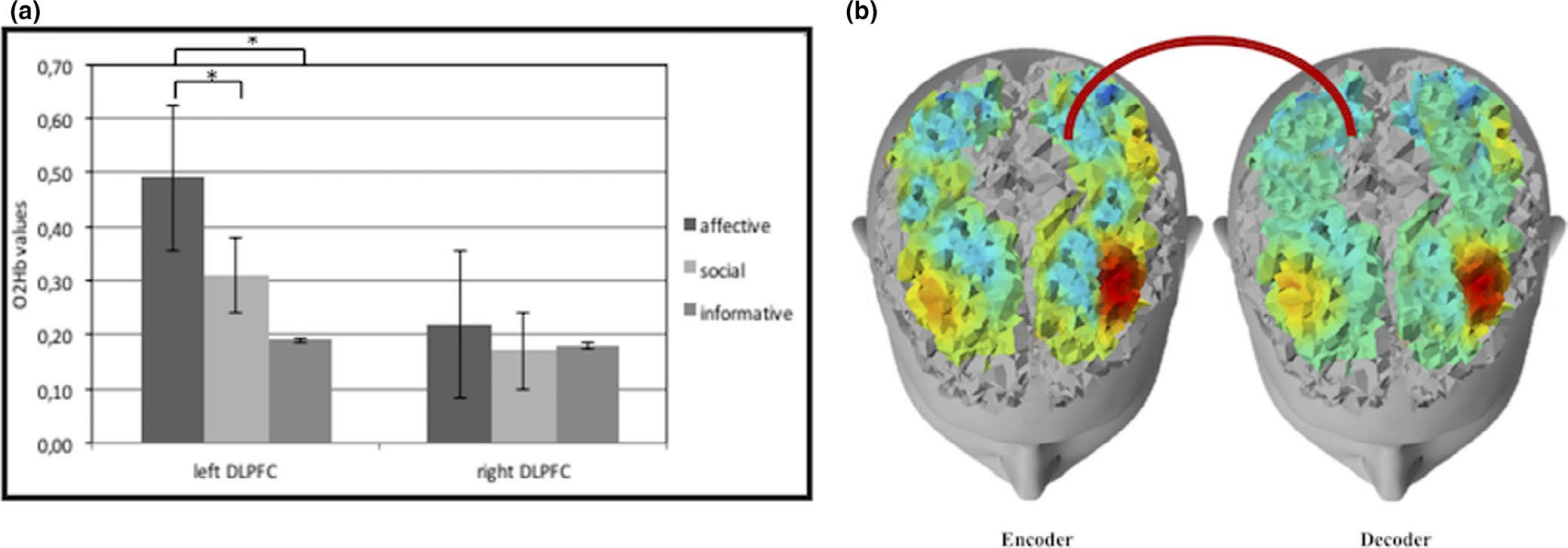
(a) Histogram of O2Hb interbrain connectivity for three different types of gestures (affective, social, and informative) in the left and right side of the DLPFC. Bars represent +‐1SE. Stars mark statistically significant (*p*<.05) pairwise comparisons. (b) Representation of O2Hb interbrain connectivity for affective gestures in DLPFC left and right side in encoder and decoder. The red color shows the increase of O2Hb interbrain connectivity in the DLPFC left side compared with the right one for affective gestures

In addition, positive gestures showed an increase of interbrain synchronization in the left DLPFC area: this effect was specific for affective gesture more than social (*F*[1,33] = 9.33; *p* < .001; *η* ^2^ = 0.35) and informative ones (*F*[1,33] = 8.21; *p* < .001; *η* ^2^ = 0.30; Figure 6a,b).

## 4 DISCUSSION

The present study aimed to investigate the brain responsiveness and interbrain correlates associated with the observation of different gestures' types during a non‐verbal interaction between encoder and decoder. In particular, the present study aimed to investigate the neural correlates underlying the observation of affective, social, and informative gestures with positive and negative valence. Specifically, in order to observe interagents' individuals brain responsiveness and brain tuning mechanisms, single‐brain and interbrain analyses were conducted.

Firstly, from the results of the single‐brain analysis, according to our hypothesis, an increase of O2Hb and a decrease of HHb activity were observed for affective gestures observation in the DLPFC and for social gestures observation in the SFG area. This result highlights the activation of specific brain areas according to the category of gesture observed.

Specifically, the greater activation of O2Hb activity in the DLPFC area for affective gestures observation may be due to the functional significance of these types of gestures aimed to transmit emotionally charged meanings and to share emotional experiences (Tomasello et al., 2005). Considering, therefore, the functional meaning of affective gestures, the increase of O2Hb activity in the DLPFC region can be related to a higher involvement of this cerebral area in emotional, prosocial, and empathic processes (Baeken et al., 2011; Balconi, Pezard, Nandrino, & Vanutelli, 2017; Kalbe et al., 2010) that can be experienced by individuals during affective gestures observation.

Moreover, DLPFC area appears to be involved in some processes that can be activated by affective gestures, such as theory of mind mechanisms, interpersonal relationships and other people's states understanding (Bavelas et al., 1992; Bressem & Müller, 2017; Calbris, 2011; Kendon, 2017; Müller, 2004, 2016). These results also appear to be confirmed by previous research that has observed an increase of frontal activity concerning emotional affective gestures observation (Peyk, Schupp, Keil, Elbert, & Junghöfer, 2009). Furthermore, DLPFC compare with SFG, FEF, DPMC areas appears to be more involved in the ability to respond motivationally to innate or learned nonverbal social cues, such as facial expressions and emotional tone in speech or gestures. Moreover, DLPFC appears to be involved in understanding and reinterpreting the meaning of a stimulus to downregulate emotional response (Gökçay & Yildirim, 2010).

Similarly, the increase of O2Hb in SFG area for social gestures observation can be interpreted in light of the functional meaning of social gestures finalized to initiate, establish, or interrupt a relationship with another individual (Bavelas et al., 1992; Kendon, 2017). In light of the functional significance of social gestures, the greater activation of O2Hb in SFG region may be because this cerebral area appears to be involved in mechanisms of behavior control and in others' intentions implementation (Crivelli & Balconi, 2017; Nakamura et al., 1998; Shima & Tanji, 2017).

Concerning this first result, it is interesting to observe how both for O2Hb and HHb values the same trend occurs in individual brain responsiveness, as evidenced by a simultaneous increase of O2Hb and decrease of HHb in the same cerebral areas according to specific gestures observation. Moreover, according to our hypothesis, an increase of O2Hb activity has emerged in the left DLPFC region during the observation of positive affective gestures. This lateralized effect confirms the theory of the dual system model of neural signatures of affective experience (Balconi et al., 2015; Davidson, 1992), which postulates that stimuli perceived by individuals as positive induce approaching behaviors and positive emotions experience, leading to a greater activation of the left frontal side; while, a more greater activation of the frontal right side results to be associated with the presentation of negative stimuli providing avoidance behaviors (Balconi & Mazza, 2009, 2010; Davidson, 1992; Harmon‐Jones, 2003). On the basis of this model, therefore, the greater activation of DLPFC on the left side could be due because positive affective gestures' observation, such as seeing one individual caressing another, elicits individuals' positive emotions.

Considering, instead, interbrain results, an increase of O2Hb interbrain connectivity in DLPFC area emerged during the observation of affective gestures; while an increase of O2Hb interbrain connectivity emerged in SFG area during the observation of social gestures. This result shows how these cerebral areas, which support emotional regulation, interaction and social understanding mechanisms (Baker, Bloom, & Davis, 2016; Kalbe et al., 2010; Liu et al., 2015; Suzuki et al., 2011), are involved in mirroring mechanisms that allow individuals to synchronize their brain responses during gestures observation (Marsh, Blair, Jones, Soliman, & Blair, 2009). Moreover, this result highlights that during affective and social gestures observation, neural synchronization and implicit coupling mechanisms occur between encoders and decoders, presupposing a sharing and a co‐representation of actions that equally involve both individuals, as if they were preparing for the implementation of a synchronized response to movement.

Furthermore, in light of this result, it emerges that an understanding of the meaning of these types of gestures occurs during gestures observation both in encoder and decoder, which leads individuals to prepare for the development of joint action. Indeed, as has been shown by previous studies, during the development of joint actions, synchronic, and diachronic mechanisms take place in individuals, increasing the implicit neural coupling and interpersonal coupling dynamics (Balconi, Fronda, & Vanutelli, 2019; Balconi, Pezard, et al., 2017).

Concerning gesture valence, instead, from interbrain connectivity an increase of O2Hb activity in the left DLPFC area has emerged concerning positive affective gestures observation. This result confirms the frontal brain asymmetry postulated by the dual system model of neural signatures of affective experience according to the presentation of positive and negative stimuli (Balconi et al., 2015; Davidson, 1992).

Finally, it is interesting to notice that the outcome of the present study did not reveal any significant differences in the brain activity of encoder and decoder during gestures observation, despite the different roles of interagents that required encoder to observe the gesture in view of future reproduction and decoder to only observe the gesture reproduced by the video without any other action. This direct combination of observation and planning of the gesture has been observed by several studies (Chong et al., 2008; Rizzolatti & Craighero, 2004; Rizzolatti et al., 2001), pointing out that actions understanding occur when the observation activate the observer motor region (Chong et al., 2008; Rizzolatti et al., 2001).

This similar neural activation has shown the involvement of the same cerebral areas during processes of gestures observation and gestures imagination and planning (Buccino, Binkofski, & Riggio, 2004; Chong et al., 2008; Coricelli et al., 2005; Gallese, 2003; Gallese et al., 1996; Rizzolatti & Craighero, 2004; Rizzolatti et al., 1996; Wilson & Knoblich, 2005). In the present study, the main frontal areas involved in encoder/decoder response may be represented as supporting mirroring mechanisms in the case of affective and social action representation, able to produce a dual resonance in both active and passive actor.

In conclusion, the present study highlighted different activation schemes underlying the observation of different types of positive and negative gestures. Furthermore, the use of hyperscanning and the implementation of interbrain analysis allowed us to underline the presence of mirroring mechanisms involved in gesture‐specific frontal regions during gestures observation and action planning, with a clear synchronization in two brains.

Despite the potential of this study, some limits may be highlighted that could be taken in consideration for future studies. Firstly, by implementing the sample size, the power of the observations obtained could be increased. Secondly, the study could be repeated using different interaction contexts for the observation of specific categories of gestures.

Thirdly, the use of other neuroscientific techniques (such as electroencephalography) could allow us to gather further data in terms of temporal evolution of the interbrain dynamics, which is useful to confirm or add new evidence to results.

Fourthly, to better generalize the present results, an ample sample size could be suggested for future investigations.

At present, power analysis supported the results as a pilot study, in the absence of population as a reference for the sample size. Fifth, future analysis could be considered the comparison between encoder and decoder during the step of gesture reproduction by encoder, in which the encoder reproduces the gesture toward the decoder who passively receives it, to investigate other neural mechanisms underlined this moment, quite different from mirroring mechanisms presenting during gestures' observation. Finally, besides mechanisms of synchrony and symmetric interbrain connectivity in the same cerebral areas, in future studies the asymmetric pattern of coupling in different cerebral areas should be explored to observe the different psychological process of the subjects during social interactions.

## CONFLICT OF INTEREST

Authors declare they have not conflict of interests.

## AUTHOR CONTRIBUTION

MB contributed to the conception and design of the study; MB wrote the first draft and each section of the manuscript. MB and GF contributed to manuscript final writing and revision, read and approved the submitted version.

## Data Availability

The data that support the findings of this study are available from the corresponding author upon reasonable request.
